# Supplementary material for: SMOC can act as both an antagonist and an expander of BMP signaling
Source: eLife. 2017 Mar 21;6:e17935. doi: 10.7554/eLife.17935 (PMC5360445; doi:10.7554/eLife.17935)
Supplement: Figure 4—source data 1. — Absorbance values obtained from pSmad immunoblots in four separate experiments. DOI: http://dx.doi.org/10.7554/eLife.17935.010 [file elife-17935-fig4-data1.docx]

Figure 4 – Source Data

|  | **Absorbance Value from Immunoblot** | | | | |
| --- | --- | --- | --- | --- | --- |
| **Experiment #** | **Control** | **BMP2** | **BMP2 + SMOC** | **BMP2 + ∆EC** | **BMP2 + EC** |
| 1 | 8.88 | 12.9 | 9.54 | 8.98 | 17.27 |
| 2 | 14.42 | 19.38 | 13.49 | 10.77 | 35.01 |
| 3 | 11.87 | 15.91 | 12.58 | 11.33 | 22.84 |
| 4 | 6.98 | 10.40 |  |  | 11.85 |
