# Supplementary material for: SMOC can act as both an antagonist and an expander of BMP signaling
Source: eLife. 2017 Mar 21;6:e17935. doi: 10.7554/eLife.17935 (PMC5360445; doi:10.7554/eLife.17935)
Supplement: Figure 6—source data 1. — The number of Luciferase positve cells in five fields of view were calculated as a percentage of the total number of cells in each field. DOI: http://dx.doi.org/10.7554/eLife.17935.014 [file elife-17935-fig6-data1.docx]

**Figure 6: Source Data**

| **Agarose/Heparan Sulfate Gel** | | | **Agarose/Heparan Sulfate/SMOC-EC Gel** | | |
| --- | --- | --- | --- | --- | --- |
| Number of Cells/field | Number of Luciferase positive cells | % Positive | Number of Cells/field | Number of Luciferase positive cells | % Positive |
| 47 | 8 | 17 | 118 | 86 | 73 |
| 65 | 11 | 17 | 81 | 35 | 43 |
| 100 | 24 | 24 | 108 | 62 | 57 |
| 108 | 20 | 18 | 35 | 34 | 97 |
| 47 | 5 | 11 | 37 | 29 | 78 |
|  |  |  |  |  |  |
